# Distinct brain network patterns in complete and incomplete spinal cord injury patients based on graph theory analysis

**DOI:** 10.1111/cns.14910

**Published:** 2024-08-26

**Authors:** Beining Yang, Haotian Xin, Ling Wang, Qunya Qi, Yu Wang, Yulong Jia, Weimin Zheng, Chuchu Sun, Xin Chen, Jubao Du, Yongsheng Hu, Jie Lu, Nan Chen

**Affiliations:** ^1^ Department of Radiology and Nuclear medicine, Xuanwu Hospital Capital Medical University Beijing China; ^2^ Beijing Key Laboratory of Magnetic Resonance Imaging and Brain Informatics Beijing China; ^3^ Department of Radiology, Beijing Chaoyang Hospital Capital Medical University Beijing China; ^4^ Department of Rehabilitation Medicine, Xuanwu Hospital Capital Medical University Beijing China; ^5^ Department of Functional Neurosurgery, Xuanwu Hospital Capital Medical University Beijing China

**Keywords:** complete spinal cord injury, functional connectivity, incomplete spinal cord injury, structural connectivity, structural–functional coupling

## Abstract

**Aims:**

To compare the changes in brain network topological properties and structure–function coupling in patients with complete spinal cord injury (CSCI) and incomplete spinal cord injury (ICSCI), to unveil the potential neurobiological mechanisms underlying the different effects of CSCI and ICSCI on brain networks and identify objective neurobiological markers to differentiate between CSCI and ICSCI patients.

**Methods:**

Thirty‐five SCI patients (20 CSCI and 15 ICSCI) and 32 healthy controls (HCs) were included in the study. Here, networks were constructed using resting‐state functional magnetic resonance imaging to analyze functional connectivity (FC) and diffusion tensor imaging for structural connectivity (SC). Then, graph theory analysis was used to examine SC and FC networks, as well as to estimate SC‐FC coupling values.

**Results:**

Compared with HCs, CSCI patients showed increased path length (*L*p), decreased global efficiency (*E*_g_), and local efficiency (*E*_loc_) in SC. For FC, ICSCI patients exhibited increased small‐worldness, clustering coefficient (*C*_p_), normalized clustering coefficient, and *E*_loc_. Also, ICSCI patients showed increased *C*_p_ and *E*_loc_ than CSCI patients. Additionally, ICSCI patients had reduced SC‐FC coupling values compared to HCs. Moreover, in CSCI patients, the SC network's *L*p and *E*_g_ values were significantly correlated with motor scores, while in ICSCI patients, the FC network's *C*_p_, *E*_loc_, and SC‐FC coupling values were related to sensory/motor scores.

**Conclusions:**

These results suggest that CSCI patients are characterized by decreased efficiency in the SC network, while ICSCI patients are distinguished by increased local connections and SC‐FC decoupling. Moreover, the differences in network metrics between CSCI and ICSCI patients could serve as objective biological markers, providing a basis for diagnosis and treatment strategies.

## Graphical abstract

The diagram of brain network construction and analysis. AAL, anatomical automatic labeling.

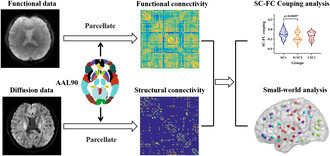

## 1 INTRODUCTION

Traumatic spinal cord injuries (SCI), caused by external factors such as accidents or falls that disrupt the normal functioning of ascending and descending neural tracts, significantly impact patients' physical, emotional, and social well‐being.^1^ The severity of the SCI plays a critical role in determining the prognostic outcomes and the potential for recovery, highlighting the importance of accurate assessment and classification of SCI.^2^ Currently, the International Standard for Neurological Classification of Spinal Cord Injury (ISNCSCI) provides a framework for classifying patients into complete SCI (CSCI) or incomplete SCI (ICSCI), based on the preservation of sacral sensorimotor functions.^3^ Such accurate categorization is crucial in devising targeted treatment and rehabilitation strategies. For instance, rehabilitation for ICSCI patients focuses on maximizing the recovery of partially retained motor and sensory functions to improve independence and quality of life.^4^, ^5^, ^6^ However, at present, due to the lack of effective treatment methods, rehabilitation for patients with CSCI tends to focus on adapting to the loss of function, preventing secondary complications, and enhancing overall quality of life through the use of assistive technologies and modifications to the living environment rather than undergoing potentially effective functional recovery treatments.^7^, ^8^, ^9^

It is crucial to note that the loss of sensorimotor function below the level of injury in patients with CSCI does not necessarily mean there are no preserved residual sensorimotor pathways. Previous studies have demonstrated the presence of sensorimotor pathway in individuals clinically classified as having CSCI, suggesting potential avenues for therapeutic intervention and recovery.^10^, ^11^ Currently, the assessment of sensory and motor function in SCI patients primarily relies on clinical functional examinations. However, these assessments can be complex and subjective, often influenced by various confounding variables. This leads to challenges in accurately detecting residual sensorimotor pathways, with studies showing that approximately 25% of patients initially diagnosed with CSCI may reclassify to ICSCI within the first year post‐injury.^12^ Therefore, given these challenges, there is a pressing need for the identification of objective neurobiological markers that can reliably differentiate between CSCI and ICSCI, to improve prognosis, inform treatment decisions, and guide the development of personalized rehabilitation plans.

Direct neurological damage, secondary impacts, and other factors following SCI contribute to variations in brain structure and function, differences that may manifest distinctly between patients with CSCI and ICSCI. Magnetic resonance imaging (MRI), acknowledged as an objective and non‐invasive method, has been validated for its effectiveness in detecting such disparities.^13^, ^14^ Notably, one study illustrated that patients with CSCI exhibited reduced functional amplitude of low‐frequency fluctuations in specific brain regions, including the right superior medial frontal gyrus and right middle frontal gyrus, in contrast to ICSCI patients.^13^ Moreover, another study has pinpointed discrepancies in the gray matter volume in primary somatosensory cortex and primary motor cortex between CSCI and ICSCI groups.^14^ However, some prior studies employing voxel‐based morphometry and diffusion tensor imaging (DTI) have not identified differences between CSCI and ICSCI patients.^15^, ^16^ Differences in previous studies may stem from a reliance solely on voxel or tract‐based spatial statistics, overlooking alterations in the patterns of connectivity between brain regions and the overarching network structure. Therefore, implementing brain network analysis could offer further insight into the mechanisms behind the changes in brain structure experienced by patients with CSCI and ICSCI.

Network analysis, leveraging the principles of graph theory, has emerged as a potent tool in disease research, providing a novel lens through which the brain's intricate structure and dynamics can be understood.^17^, ^18^, ^19^ By investigating the brain's topological properties, network analysis aims to uncover the overall patterns of connectivity, and reveal the underlying physiological or disease processes from the network and information processing perspectives. In SCI research, network analysis has illuminated significant changes in the brain's network organization.^17^, ^18^ For example, analyses of functional connectivity (FC) in individuals with chronic CSCI have identified a marked decrease in local efficiency (*E*_loc_), suggesting disruptions in the brain's local information processing capabilities.^18^ Similarly, our group's DTI study on structural connectivity (SC) in pediatric patients with thoracolumbar SCI has shown alterations in network parameters, including reduced shortest path length (*L*p) and normalized path length (*λ*), alongside increased global efficiency (*E*_g_).^17^ Yet, discrepancies exist in the previous studies, with some studies reporting no significant differences in global indicators between SCI patients and healthy controls (HCs) in SC networks.^20^, ^21^ This difference may originate from the studies' reliance solely on single imaging methods, which might not fully capture the entire scope of brain network alterations in SCI patients, including the interactions between the SC and FC networks. Specifically, SC captures the anatomical pathways across the brain and FC measures the correlation between the activity of brain regions.^22^ The interplay between SC and FC is critical for understanding the brain's adaptability, functionality, and response to injuries or diseases.^23^, ^24^, ^25^

The concept of SC‐FC coupling, an integrative measure that combines information from both SC and FC networks, has been increasingly applied in the study of neuropsychological diseases, including stroke, cognitive impairment, and schizophrenia.^26^, ^27^, ^28^ This approach may have the potential to provide a more comprehensive understanding of SCI. Therefore, by employing network analysis and SC‐FC coupling methods in SCI research, we can gain deeper insights into the complex alterations in brain networks associated with both CSCI and ISCI.

In this study, we aim to investigate the topological patterns alternations of SC, FC network, and SC‐FC coupling, as well as their correlations with clinical symptoms, in order to reveal the underlying mechanisms of brain structural and functional changes in patients with CSCI and ICSCI. Through this research, we hope to offer objective biomarkers for diagnosis and support the advancement of therapeutic and rehabilitation strategies.

## 2 PARTICIPANTS AND METHODS

After receiving approval from the ethics committee of Xuanwu Hospital and securing written informed consent from participants, this study included 37 SCI patients confirmed at Xuanwu Hospital, along with 32 age‐ and gender‐matched HCs. All SCI patients underwent evaluations for motor and sensory scores, level of lesion, and ASIA classification according to the ISNCSCI scale, conducted by two seasoned neurology clinicians, each with over 25 years of experience. An injury was categorized as CSCI if there was a total absence of sensory and motor function at the S4‐5 sacral segments. Consequently, the SCI patients were bifurcated into two groups: 16 in the ICSCI group and 21 in the CSCI group. Neuropathic pain assessments were performed using the Visual Analog Scale (VAS) by an experienced rehabilitation clinician. The inclusion criteria for ICSCI participants are as follows: (1) Traumatic SCI (resulted from traffic accidents, falls, sports injuries, violence, or natural disasters); (2) right‐handedness, ASIA B‐D grade, and with an injury duration of more than 2 months; (3) no abnormalities found in the brain on conventional MRI; (4) no history of other neurological diseases, inherited family diseases, head trauma, or mental illness; (5) no record of drug or alcohol abuse; and (6) qualified image quality for data processing. The inclusion criteria for CSCI participants are as follows: (1) traumatic SCI (resulted from traffic accidents, falls, sports injuries, violence, or natural disasters); (2) right‐handedness, ASIA A grade, and with an injury duration of more than 2 months; (3) no abnormalities found in the brain on conventional MRI; (4) no history of other neurological diseases, inherited family diseases, head trauma, or mental illness; (5) no record of drug or alcohol abuse; and (6) qualified image quality for data processing. The inclusion criteria for the HCs group are as follows: (1) right‐handedness, age, and sex matched for the SCI group; (2) no abnormalities found in the brain on conventional MRI; (3) no history of neurological diseases; (4) no record of drug or alcohol abuse; and (5) qualified image quality for data processing. After examination by two radiologists, one ICSCI patient and one CSCI patient were excluded due to severe head motion (>2.5 mm). Finally, 15 ICSCI patients, 20 CSCI patients, and 32 HCs were included in this study. The detailed clinical data of 15 ICSCI patients and 20 CSCI patients are shown in Table 1.

**TABLE 1:** Clinical data of 20 CSCI patients and 15 ICSCI patients.

Patients' ID	Gender	Age	Duration (months)	Etiology	Motor scores (0–100)	Sensory scores (0–224)	VAS scores	Level of lesion (neurological level)	ASIA grade
1	M	50	12	Violence	24	80	9	C7	A
2	F	16	21	Traffic accident	24	128	0	C6	A
3	M	42	108	Violence	56	144	9	L4	A
4	M	38	84	Fall	56	148	9	L4	A
5	M	52	12	Traffic accident	50	152	8	L2	A
6	M	50	108	Violence	50	160	9	L4	A
7	M	55	36	Traffic accident	50	188	6	L5	A
8	M	67	132	Traffic accident	50	152	6	L4	A
9	M	52	348	Violence	50	144	8	L5	A
10	M	58	108	Violence	50	132	9	L4	A
11	M	34	192	Traffic accident	50	136	8	L4	A
12	M	53	192	Fall	50	96	9	T6	A
13	F	28	18	Stab	50	152	8	L1	A
14	M	31	120	Traffic accident	50	144	8	T12	A
15	M	33	18	Traffic accident	51	128	9	T11	A
16	M	49	48	Fall	50	156	8	T12	A
17	M	38	24	Violence	50	144	0	T8	A
18	F	14	48	Fall	50	124	0	T10	A
19	F	13	103	Traffic accident	50	128	0	T7	A
20	F	13	18	Backbend	50	164	9	T12	A
21	M	40	192	Fall	60	142	8	C4	B
22	M	56	396	Traffic accident	58	148	8	L3	C
23	F	37	36	Fall	64	188	0	L1	C
24	M	65	6	Fall	54	144	8	T12	C
25	M	42	72	Violence	60	144	0	T11	C
26	M	10	5	Traffic accident	80	113	6	C5	C
27	F	55	9	Violence	90	152	6	C5	D
28	M	57	84	Fall	80	204	5	C5	D
29	M	71	12	Violence	70	204	9	C6	D
30	M	60	36	Fall	82	200	10	L5	D
31	M	30	60	Violence	74	190	4	L3	D
32	F	34	12	Fall	70	160	0	L4	D
33	F	28	7	Violence	86	172	8	L5	D
34	F	40	144	Traffic accident	95	182	9	L5	D
35	F	13	204	Backbend	50	152	0	T12	D

*Note*: The lesion level is defined as the lowest neurological level where sensory or motor function remains intact. ASIA impairment scale: A: complete no sensory or motor function is preserved in sacral segments S4–S5; B: incomplete sensory but not motor function is preserved below the neurological level and extends through sacral segments S4–S5; C: incomplete—motor function is preserved below the neurological level, and more than half of the key muscles below the neurological level have a muscle grade of <3; D: incomplete—motor function is preserved below the neurological level, and at least half of the key muscles below the neurological level have a muscle grade of >3. Sensory scores are determined by summing the segmental assessments of light touch and pinprick sensations.

Abbreviations: ASIA, American Spinal Injury Association; CSCI, complete spinal cord injury; F, female; ICSCI, incomplete spinal cord injury; M, male; VAS, Visual Analog Scale.

### 2.1 MRI data acquisition

MRI images were acquired using a 3.0 T Siemens scanner (Erlangen, Germany) equipped with a 12‐channel phased‐array head coil. To reduce head movement, participants were provided with earplugs and MRI‐compatible goggles and instructed to remain still. Axial T2‐weighted Turbo Spin Echo and axial fluid‐attenuated inverse recovery sequence images were used to identify any visible encephalopathy, assessed by two radiologists with extensive experience, Dr. Chen (27 years) and Dr. Lu (25 years). The scanning protocol included the following:

1. High‐resolution T1‐weighted structural images captured with a sagittal 3D T1‐weighted magnetization‐prepared rapid gradient‐echo sequence. The settings were as follows: TR = 1800 ms, TE = 2.13 ms, inversion time = 1100 ms, flip angle = 9°, field of view = 256 × 256 mm, 192 slices, slice thickness = 1 mm, matrix = 256 × 256, and voxel size = 1 × 1 × 1 mm^3^, with a total scan duration of 4 min and 55 s.
2. DTI scans were conducted with a spin‐echo echo‐planar sequence using 64 noncollinear diffusion directions. Parameters included TR = 13,200 ms, TE = 90 ms, b‐value = 1000 s/mm^2^, flip angle = 90°, acquisition matrix = 128 × 128, field of view = 256 × 256 mm^2^, 70 slices, slice thickness = 2 mm, voxel size = 2 × 2 × 2 mm^3^, and with a scan duration of 14 min and 59 s.
3. Resting‐state functional MRI (rs‐fMRI) images were obtained through an echo‐planar imaging (EPI) sequence with 35 axial slices (slice thickness = 3 mm, inter‐slice gap = 1 mm), TR = 2000 ms, TE = 30 ms, flip angle = 9°, FOV = 220 × 220 mm^2^, and a matrix of 64 × 64. These settings resulted in an isotropic voxel size of 3.4 × 3.4 × 3.0 mm^3^, covering the acquisition over 6.08 min with 180 volumes.

Participants were instructed to relax, close their eyes, stay awake, breathe evenly, and refrain from engaging in any tasks or systematic thinking during the scan.

### 2.2 Preprocessing of DTI data

The DTI data in this study were preprocessed using the FSL software package (version 4.1, https://fsl.fmrib.ox.ac.uk/fsl/fslwiki/). First, we converted the data from Digital Imaging and Communications in Medicine (DICOM) format to Neuroimaging Informatics Technology Initiative (NIfTI) format. Then, the process involved estimating a brain binary mask from the b0 image, correcting for head movement and eddy current distortions with FSL's eddy_correct tool using the b0 image as reference, and removing non‐brain tissue with the BET tool in FSL. Participants with head movements exceeding 2.5 mm translation or 3° rotation were excluded. Finally, we fitted a diffusion tensor model to the data to calculate voxel‐specific parameters like fractional anisotropy (FA) and mean diffusivity using the Diffusion Toolkit (www.trackvis.org). For whole‐brain fiber tractography, we utilized deterministic tracing with the Diffusion Toolkit software (version 0.6.3, https://trackvis.org/dtk/), based on the fiber assignment by continuous tracking algorithm. Tracking was terminated when FA fell below 0.15 or the deflection angle exceeded 45°.^29^, ^30^

### 2.3 Preprocessing of rs‐fMRI data

The rs‐fMRI data preprocessing was conducted using the GRETNA toolbox (version 2.0, https://github.com/sandywang/GRETNA), based on SPM 12 in MATLAB 2016b. The steps involved in preprocessing are outlined as follows: initially, the raw data were converted from DICOM to NIfTI format to ensure compatibility with processing tools. Following this, the first 10 time‐point volumes were removed to eliminate initial signal instability, and slice‐timing adjustments were made to the remaining 170 volumes to correct acquisition timing discrepancies. Motion correction protocols were then applied, and participants with head movements exceeding 2.5 mm (translation) or 3° (rotation) were excluded to maintain data integrity. Then, the data were standardized to Montreal Neurological Institute (MNI) space using the EPI template, with a specified voxel size of 3 × 3 × 3 mm^3^. Covariates including Friston‐24 head motion parameters, white matter, and cerebrospinal fluid signals were regressed out from the data. Temporal filtering within the frequency range of 0.01–0.08 Hz was applied to focus on relevant physiological fluctuations, followed by detrending to remove any linear or nonlinear drift in the signal, preparing the data for accurate and reliable analysis.

### 2.4 Network reconstruction

In this research, the nodes of both structural and functional brain networks were segmented into 90 regions of interest (ROIs)—45 for each hemisphere—using the Anatomical Automatic Labeling (AAL) Atlas through an automated anatomical labeling algorithm. For SC networks, we constructed brain networks for each participant using DTI tractography, using the PANDA package (version 1.3.1, https://www.nitrc.org/projects/panda/).^30^ Initially, brain‐extracted T1‐weighted images were aligned with their b0 images in native diffusion space via affine transformation. Subsequently, these T1‐weighted images were nonlinearly registered to the MNI space, and the transformation parameters from above two steps were inverted to transpose the AAL atlas from MNI space back to each participant's native diffusion tensor space. Therefore, the individual cerebrum was partitioned into 90 nodes based on the AAL atlas within its native space. To mitigate the inclusion of false‐positive connections, an edge between two nodes was established only if at least three fiber tracts were detected between them.^17^, ^27^, ^31^ For each participant, the average FA of the connecting fibers between nodes was used as the edge weight in the structural network.

For FC networks, we constructed brain networks for each participant from rs‐fMRI data using the GRETNA toolbox, with nodes corresponding to the 90 ROIs delineated by the AAL atlas. Edges were defined by the Pearson correlation coefficient between the average time series of each node, resulting in a symmetric 90 × 90 functional network matrix. Negative correlations within the functional connectivity network were excluded due to their ambiguous physiological relevance.^32^, ^33^, ^34^

### 2.5 Graph theory analysis

The GRETNA toolbox was employed to evaluate the graph‐theoretical metrics of both structural and functional brain networks. In our analysis, we set a sparsity threshold for the functional matrices in line with previous studies (ranging from 0.1 to 0.34, incremented by 0.01), and for structural matrices, we initialized a specific matrix element value, starting the threshold sequence at 0.^17^, ^35^, ^36^ To delineate the comprehensive architecture of these networks, we analyzed seven global metrics: small‐worldness (*σ*), the normalized clustering coefficient (*γ*), clustering coefficient (*C*_p_), *λ*, *L*p, *E*_g_, and *E*_loc_. To calculate *γ* and *λ*, we generated 5000 matched random networks for the SC network and 100 matched random networks for the FC network to compare with the real network's *C*_p_ and *L*p. Furthermore, for a more nuanced comparison between groups, we calculated the area under the curve (AUC) across various sparsity levels in FC. This approach provides a detailed measure of the topological organization of brain networks, circumventing the limitations inherent in selecting a single threshold.

### 2.6 SC‐FC coupling analysis

In this study, we utilized a SC‐FC coupling analysis technique that is consistent with methodologies described in prior research.^29^, ^36^ The process began with the extraction of nonzero edges from the SC matrix, resulting in a vector representing structural connectivity measures. This vector was subsequently normalized to fit a Gaussian distribution. Subsequently, we apply a Fisher‐*z* transformation to the FC matrix and extract the edges corresponding to the SC matrix, forming a vector of FC measures. The final step was the calculation of the Pearson correlation coefficient between these vectors, enabling the quantification of network coupling metrics for each participant.

### 2.7 Statistical analysis

First, we analyzed the group differences in sex using the chi‐square test. Next, the Kolmogorov–Smirnov test was employed to assess the normality of the continuous variable. For normally distributed demographic variables, one‐way analysis of variance or a two‐sample *t*‐test was conducted to examine group effects. For demographic variables that were not normally distributed, the Wilcoxon rank sum test was applied. Analysis of covariance (ANCOVA) was applied to compare SC and FC network metrics across the three groups, considering age and gender as covariates, and was complemented by post hoc tests, specifically focusing on the AUC for all FC network metrics. ANCOVA was also used to evaluate differences in SC‐FC coupling across the three groups, followed by subsequent post hoc testing. Furthermore, partial correlation analysis investigated associations between brain network characteristics (SC, FC, and SC‐FC coupling) and clinical outcomes (motor scores, sensory scores, VAS scores), adjusting for age and gender as covariates. All statistical analyses were performed using SPSS version 23.0 (IBM Inc., Armonk, NY, USA), with a significance threshold set at *p* < 0.05 for all tests.

## 3 RESULTS

### 3.1 Demographic and clinical characteristics

No significant differences were observed in age (*p* = 0.527) and gender (*p* = 0.305) among the CSCI, ICSCI, and HCs groups. Among SCI patients, there were no significant differences in injury durations (*p* = 0.341) and VAS scores (*p* = 0.240) in CSCI and ICSCI groups. However, CSCI patients presented with lower motor (48.05 ± 8.43 vs. 71.53 ± 13.74) and sensory scores (140.00 ± 27.39 vs. 166.33 ± 23.40) than those in the ICSCI group. Further details on these demographic and clinical distinctions are provided in Table 2.

**TABLE 2:** Demographic and clinical characteristics of CSCI, ICSCI patients, and HCs.

Demographic	CSCI (*N* = 20)	ICSCI (*N* = 15)	HCs (*N* = 32)	Statistics	*p‐*Value
Age (years)	39.3 ± 16.28	42.53 ± 18.09	44.13 ± 12.17	0.647	0.527^†^
Gender (male/female)	15/5	9/6	26/6	1.208	0.305^a^
Injury duration (month)	12–348	5–396	NA	−0.952	0.341^║^
Motor scores	48.05 ± 8.43	71.53 ± 13.74	NA	−4.820	<0.001^║^
Sensory scores	140.00 ± 27.39	166.33 ± 23.40	NA	−3.064	0.004^‡^
VAS scores	6.60 ± 3.50	5.40 ± 3.72	NA	−1.237	0.240^║^

*Note*: Data are means ± standard deviations.

Abbreviations: CSCI, complete spinal cord injury; HCs, healthy controls; ICSCI, incomplete spinal cord injury; NA, not applicable; VAS, Visual Analog Scale.

^†^ *p*‐Value was obtained with one‐way analysis of variance.

^‡^ *p*‐Value was obtained with the two‐sample *t*‐test.

^║^ *p*‐Value was obtained by using the Wilcoxon rank sum test.

^a^ *p*‐Value was obtained with the Chi‐square test.

### 3.2 Network metrics comparison for SC

**FIGURE 1:**
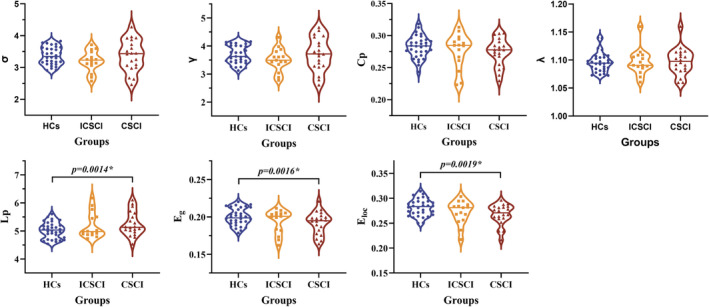
Comparison of network properties in SC network among the HCs group, ICSCI group, and CSCI group. *Significant group differences, *p* < 0.05. *C*_p_, clustering coefficient; CSCI, complete spinal cord injury; *E*_g_, global efficiency; *E*_loc_, local efficiency; HCs, healthy controls; ICSCI, incomplete spinal cord injury; *L*p, characteristic path length; SC, structural connectivity; *γ*, normalized clustering coefficient; *λ*, normalized characteristic path length; *σ*, small‐worldness.

The CSCI, ICSCI, and HCs groups exhibited small‐world properties in SC networks, characterized by *γ* > 1, *λ* ≈ 1, and *σ* > 1. Compared to HCs, the CSCI group showed significantly decreased *E*_g_ (0.19 ± 0.015 vs. 0.20 ± 0.011) and *E*_loc_ (0.27 ± 0.021 vs. 0.28 ± 0.017) and increased *L*p (5.25 ± 0.425 vs. 5.02 ± 0.288). However, no significant differences in *E*_g_ (*p* = 0.11, *p* = 0.45), *E*_loc_ (*p* = 0.31, *p* = 0.15), and *L*p (*p* = 0.30, *p* = 0.13) were detected between the ICSCI group and the other two groups (CSCI and HCs). Additionally, there were no significant differences in *σ* (*p* = 0.152), *γ* (*p* = 0.181), *C*_p_ (*p* = 0.249), and *λ* (*p* = 0.711) across the three groups (Figure 1 and Table 3).

**TABLE 3:** Brain network properties of SC and FC networks in CSCI, ICSCI patients, and HCs.

Network property	CSCI (*N* = 20)	ICSCI (*N* = 15)	HCs (*N* = 32)	Statistics	*p*‐Values
SC network					
Sigma (*σ*)	3.37 ± 0.493	3.20 ± 0.330	3.37 ± 0.279	1.946	0.152
Gamma (*γ*)	3.70 ± 0.591	3.51 ± 0.407	3.69 ± 0.331	1.756	0.181
*C*_p_	0.27 ± 0.021	0.28 ± 0.027	0.28 ± 0.017	1.422	0.249
Lambda (*λ*)	1.10 ± 0.024	1.10 ± 0.023	1.10 ± 0.017	0.343	0.711
*L*p	5.25 ± 0.425	5.19 ± 0.456	5.02 ± 0.288	4.771	0.012*
*E*_g_	0.19 ± 0.015	0.20 ± 0.014	0.20 ± 0.011	5.191	0.008*
*E*_loc_	0.27 ± 0.021	0.27 ± 0.023	0.28 ± 0.017	4.560	0.014*
FC network (AUC)					
Sigma (*σ*)	0.37 ± 0.059	0.42 ± 0.092	0.36 ± 0.062	3.497	0.036*
Gamma (*γ*)	0.43 ± 0.071	0.48 ± 0.099	0.41 ± 0.072	3.987	0.024*
*C*_p_	0.10 ± 0.013	0.11 ± 0.012	0.10 ± 0.010	4.893	0.011*
Lambda (*λ*)	0.27 ± 0.008	0.27 ± 0.011	0.27 ± 0.012	0.022	0.978
*L*p	0.71 ± 0.083	0.67 ± 0.047	0.70 ± 0.064	1.877	0.162
*E*_g_	0.08 ± 0.010	0.09 ± 0.006	0.08 ± 0.008	2.259	0.113
*E*_loc_	0.12 ± 0.048	0.13 ± 0.012	0.13 ± 0.011	3.888	0.026*
SC‐FC Coupling	−0.14 ± 0.076	−0.16 ± 0.091	−0.10 ± 0.083	4.015	0.023*

*Note*: Data are means ± standard deviations.

Abbreviations: *C*_p_, clustering coefficient; CSCI, complete spinal cord injury; *E*_g_, global efficiency; *E*_loc_, local efficiency; FC, functional connectivity; HCs, healthy controls; ICSCI, incomplete spinal cord injury; *L*p, characteristic path length; SC, structural connectivity; *γ*, normalized clustering coefficient; *λ*, normalized characteristic path length; *σ*, small‐worldness.

* Significant group differences, *p* < 0.05.

### 3.3 Network metrics comparison for FC

**FIGURE 2:**
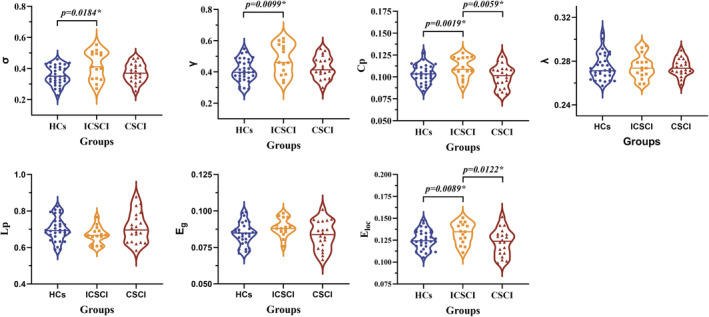
Comparison of network properties in FC network among the HCs group, ICSCI group, and CSCI group. *Significant group differences, *p* < 0.05. *C*_p_, clustering coefficient; CSCI, complete spinal cord injury; *E*_g_, global efficiency; *E*_loc_, local efficiency; FC, functional connectivity; HCs, healthy controls; ICSCI, incomplete spinal cord injury; *L*p, characteristic path length; *γ*, normalized clustering coefficient; *λ*, normalized characteristic path length; *σ*, small‐worldness.

All groups—CSCI, ICSCI, and HCs—demonstrated small‐world characteristics in functional connectivity (FC) networks, indicated by *γ* > 1, *λ* ≈ 1, and *σ* > 1. The *σ* values were notably higher in the ICSCI group compared to the HCs group (0.42 ± 0.092 vs. 0.36 ± 0.062), with no differences noted between the HCs and CSCI groups (*p* = 0.31), as well as the CSCI groups and ICSCI groups (*p* = 0.08). Furthermore, the ICSCI group showed a significant increase in *C*_p_ (0.11 ± 0.012 vs. 0.10 ± 0.010 vs. 0.10 ± 0.013) and *E*_loc_ (0.13 ± 0.012 vs. 0.13 ± 0.011 vs. 0.12 ± 0.048) compared to both HCs and CSCI groups, with *σ* (0.42 ± 0.092 vs. 0.36 ± 0.062) and *γ* (0.48 ± 0.099 vs. 0.41 ± 0.072) also being significantly higher in the ICSCI group than in the HCs group. No differences in *C*_p_ (*p* = 0.50) and *E*_loc_ (*p* = 0.51) were found between HCs and CSCI groups (*p* > 0.05). Furthermore, there were no significant differences in *λ* (*p* = 0.978), *L*p (*p* = 0.162), and *E*_g_ (*p* = 0.113) among the CSCI, ICSCI, and HCs groups (Figure 2 and Table 3).

### 3.4 SC‐FC coupling comparison

**FIGURE 3:**
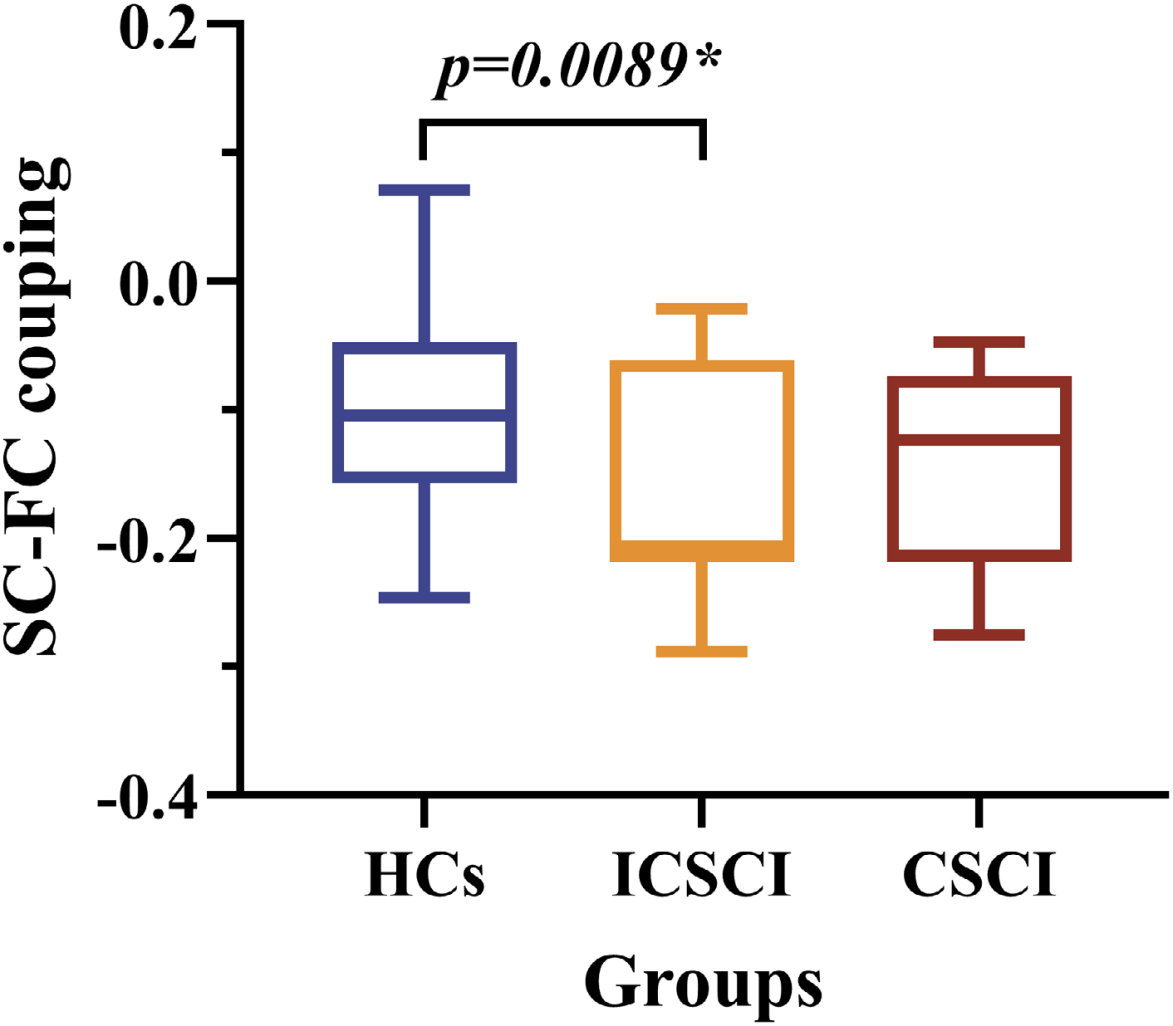
Comparison of SC‐FC coupling among the HCs group, ICSCI group, and CSCI group. *Significant group differences, *p* < 0.05. CSCI, complete spinal cord injury; FC, functional connectivity; HCs, healthy controls; ICSCI, incomplete spinal cord injury; SC, structural connectivity.

A significant reduction in structural–functional (SC‐FC) coupling was observed in the ICSCI group (−0.16 ± 0.091) in comparison to HCs (−0.10 ± 0.083) (Figure 3 and Table 3). There were no significant differences in SC‐FC coupling between the CSCI and HCs groups or between the CSCI (*p* = 0.13) and ICSCI groups (*p* = 0.63).

### 3.5 Relationships between clinical performance and network property

**FIGURE 4:**
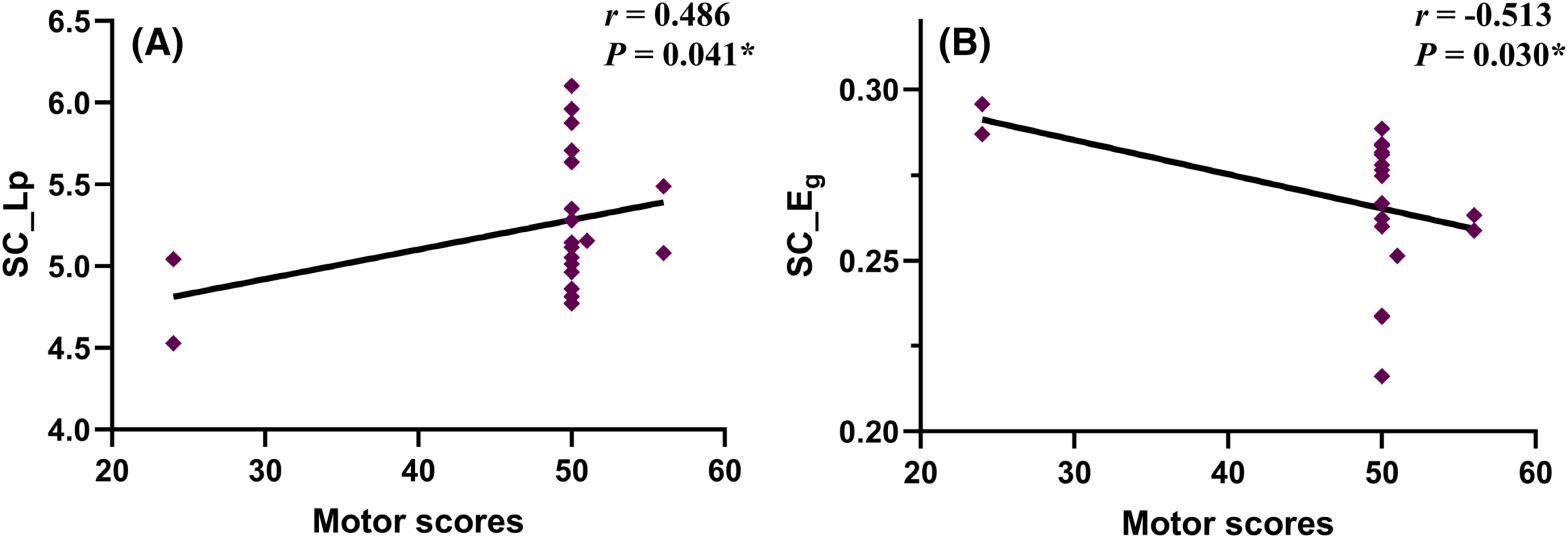
Correlations between the network properties and clinical variables in CSCI patients. (A) In the SC network, significant positive correlation between *L*p values and motor scores was observed in the CSCI group (*r* = 0.486, *p* = 0.041). (B) In the SC network, significant negative correlation was identified between *E*_g_ values and motor scores within the CSCI group (*r* = −0.513, *p* = 0.030). *Significant group differences, *p* < 0.05. CSCI, complete spinal cord injury; SC, structural connectivity; *L*p, characteristic path length; *E*_g_, global efficiency.

**FIGURE 5:**
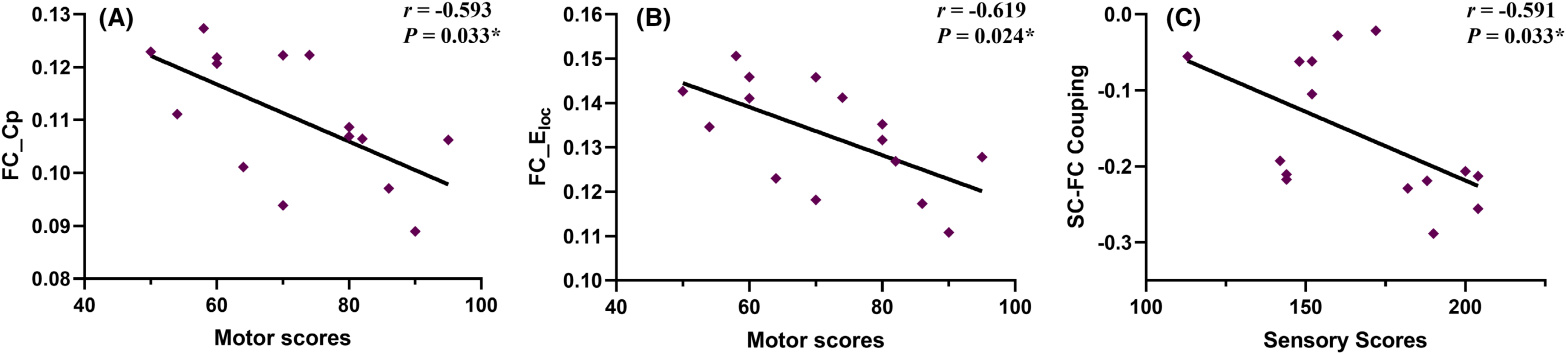
Correlations between the network properties and clinical variables in ICSCI patients. (A) In the ICSCI group, *C*_p_ values in the FC network show significant negative correlations with motor scores(*r* = −0.593, *p* = 0.033). (B) In the ICSCI group, *E*_loc_ values in the FC network show significant negative correlations with motor scores(*r* = −0.619, *p* = 0.024). (C) In the ICSCI group, SC‐FC coupling values in the FC network show significant negative correlations with sensory scores (*r* = −0.591, *p* = 0.033). *Significant group differences, *p* < 0.05. *C*_p_, clustering coefficient; *E*_loc_, local efficiency; FC, functional connectivity; ICSCI, incomplete spinal cord injury; SC, structural connectivity.

In the CSCI patient group, positive correlations were observed between motor scores and *L*p in the SC network (*r* = 0.486, *p* = 0.041; Figure 4A). Conversely, *E*_g_ in the SC network demonstrated a negative correlation with motor scores (*r* = −0.513, *p* = 0.030; Figure 4B). In the ICSCI group, motor scores were negatively correlated with the *C*_p_ (*r* = −0.593, *p* = 0.033; Figure 5A) and *E*_loc_ (*r* = −0.619, *p* = 0.024; Figure 5B) in the FC network, while sensory scores showed a negative association with SC‐FC coupling values (*r* = −0.591, *p* = 0.033; Figure 5C). No additional significant correlations were found between motor/sensory scores and the altered network properties within the SC and FC networks for both CSCI and ICSCI groups (*p* > 0.05).

## 4 DISCUSSION

In this study, we employed multimodal MRI techniques to investigate abnormalities in SC and FC networks, as well as their coupling, in patients with CSCI and ICSCI. Our findings highlighted significant reductions in *E*_g_ and *E*_loc_, alongside an increase in *L*p within SC networks of the CSCI group. Conversely, the ICSCI group demonstrated significant enhancements in *σ*, *C*_p_, *γ*, and *E*_loc_ within FC networks, with ICSCI patients showing notably higher *C*_p_ and *E*_loc_ compared to those with CSCI. Furthermore, a marked decline in SC‐FC coupling was noted in the ICSCI group. Importantly, our research identified correlations between significantly altered network metrics and sensory/motor scores in both CSCI and ICSCI groups. Therefore, this study unveils novel insights into the distinct effects of CSCI and ICSCI on brain networks, offering potential imaging biomarkers for diagnosis and contributing to the development of therapeutic and rehabilitation strategies.

### 4.1 Network metrics comparison for SC

In this study, we discovered small‐world network topology across three groups within the SC networks, essential for the brain's efficient information processing. Notably, deviations in certain small‐world and network efficiency metrics were observed in the cohort of patients with CSCI. Specifically, in comparison to HCs, patients with CSCI exhibited a significant increase in the *L*p value among small‐world indicators, along with notable decreases in *E*_g_ and *E*_loc_ values within network efficiency metrics, deepening our understanding of the neural mechanisms of CSCI. The elevation in *L*p, coupled with the reduction in *E*_g_, underscores a diminished efficiency within the SC network in CSCI patients.^37^ This pattern suggests potential decreased brain WM myelination in SCI patients, which is consistent with previous DTI studies.^38^, ^39^ We speculate that this may indicate a decrease in brain WM myelination due to the complete interruption of ascending and descending fibers at the site of SCI. Furthermore, these observations contrast with our earlier findings in pediatric SCI patients, who showed decreased *L*p and increased *E*_g_.^17^ The reason for the differing directions of SC network changes between adults and children with SCI remains uncertain. One hypothesis suggests that the impact of SCI on the brain varies across developmental stages.^40^ During childhood, the brain and nervous system are in critical developmental phases and may exhibit greater resilience to injury. In contrast, the adult brain, being more mature, might be more susceptible to structural and functional degradation following injury. Another consideration is the presence of distinct pathophysiological characteristics between pediatric and adult SCI cases, potentially influenced by differences in injury type and location, leading to varied mechanisms of brain WM network reorganization.^41^ These theories underscore the need for further investigation, including longitudinal studies spanning from childhood to adulthood in SCI patients.

The *E*_loc_ metric measures how well nodes are interconnected with their adjacent regions. A reduction in *E*_loc_ indicates a lower fault tolerance in the network, where connectivity problems in one area can significantly impact its links to related regions.^42^ Moreover, our analysis found no changes in the topological properties of the SC network among patients with ICSCI, potentially due to the preservation of some nerve fibers connecting the brain and spinal cord, maintaining the integrity of brain WM fiber connections. Overall, our results reveal reductions in both global and local connectivities within the SC network of CSCI patients, providing new insights into the neurobiological mechanisms of CSCI. Although no topological differences were directly observed between CSCI and ICSCI patients, the alterations identified in CSCI cases could help differentiate between these groups.

### 4.2 Network metrics comparison for FC

Despite the common small‐world topology across FC networks, significant differences were observed among the three groups in certain small‐world and network efficiency metrics. Specifically, we noted increases in *σ*, *C*_p_, and *γ* among small‐world metrics, and an elevation in *E*_loc_ within the ICSCI patient group, reflecting enhanced brain network efficiency. First, the elevated *σ* value indicates that the FC network in ICSCI patients possesses stronger small‐world characteristics, signifying an improved balance between information segregation and integration.^43^ This may be a compensatory change due to reduced afferent and efferent connections in the sensorimotor areas of ICSCI. Additionally, the *C*_p_ and *γ* metrics quantify the brain's functional segregation capability, with higher values denoting greater efficiency in information processing within the network.^31^ The increase in *E*_loc_ further suggests increased connectivity among adjacent brain regions in ICSCI patients. We speculate that these alterations could be associated with the diminished, yet not entirely absent, sensorimotor function in ICSCI patients, consequently increasing functional connectivity among brain regions, as supported by a prior fMRI investigation conducted by Min, Yu‐Sun et al.^44^ Contrary to a prior fMRI investigation reporting decreased *E*_loc_ in CSCI patients, our findings show no FC network abnormalities in CSCI patients, a discrepancy possibly attributable to differences in the injury segments.^18^

Compared to ICSCI patients, CSCI patients displayed significantly lower *C*_p_ and *E*_loc_ values within the FC network, although the underlying mechanisms remain elusive. One plausible interpretation is the heightened neuroplasticity observed in ICSCI patients relative to those with CSCI.^45^ This augmented neuroplasticity in ICSCI individuals likely facilitates increased functional reorganization among brain regions, thereby elevating *C*_p_ and *E*_loc_ values within the FC network. Crucially, these metrics may serve as objective neurobiological markers, aiding in the differentiation between CSCI and ICSCI patients and in the formulation of targeted therapeutic and rehabilitative strategies to enhance the quality of life for SCI individuals.

### 4.3 SC‐FC coupling comparison

Several studies have demonstrated the interaction and mutual influence between SC and FC networks.^23^, ^24^, ^25^ Specifically, the density of WM fiber bundles and the strength of connections across different brain regions can influence the formation and stability of functional networks. Conversely, the activity within these functional networks can affect the development and remodeling of WM networks. This reciprocal interaction is foundational for the brain's physiological functions and offers a crucial perspective for understanding various neurological disorders. Recently, disruptions in SC‐FC coupling have been significantly associated with the onset of numerous neurological conditions, such as stroke, cognitive impairment, and schizophrenia.^26^, ^27^, ^28^ In our research, we noted a reduction in SC‐FC coupling among patients with ICSCI, without significant differences observed between the CSCI group and the HCs group. The decreased SC‐FC coupling in ICSCI patients may indicate a loss of coherence between structural and functional networks, possibly reflecting the brain's self‐reorganization in response to SCI to adapt to a new physiological state, which is crucial for maintaining incomplete sensory and motor functions. Furthermore, this insight helps elucidate the distinct neurobiological mechanisms underlying CSCI and ICSCI, providing a theoretical basis for the development of objective indicators.

### 4.4 Relationships between clinical performance and network property

In patients with CSCI, we observed a significant positive correlation between motor scores and *L*p of the SC network, as well as a significant negative correlation with *E*_g_ of SC network. These findings suggest that patients with better motor function likely experience more pronounced changes in SC networks, indicating that the optimization and adjustment of SC network may be key factors in improving motor function in CSCI patients. In patients with ICSCI, *C*_p_ values and *E*_loc_ values of FC networks exhibited significant negative correlations with motor scores. This negative correlation suggests that excessive local clustering in FC networks might hinder motor recovery by reducing network flexibility and plasticity. Additionally, SC‐FC coupling values were significantly negatively correlated with sensory scores, further supporting the notion that a certain degree of decoupling may enable the brain network to adapt to injury by creating new functional pathways or reorganizing existing connections, thereby facilitating sensory function recovery. Moreover, the significant correlations observed underscore the efficacy of these network metrics as objective biological markers for assessing the potential for sensory‐motor function recovery in CSCI and ICSCI patients.

### 4.5 Limitations

First, the sample size in our study is relatively small, consisting of participants with a wide range of injury durations. Future research should include a larger sample size and aim for a narrower range of injury duration to enhance the robustness of the findings. Additionally, this study adopts a cross‐sectional design, which limits our ability to elucidate the mechanisms underlying brain network changes in CSCI and ICSCI over time. Future investigations should employ longitudinal designs to further explore these mechanisms and validate the significance of these metrics as objective biomarkers. Lastly, the current research predominantly emphasizes the global properties of brain networks and the overall coupling between SC and FC, inadvertently overlooking the spatial heterogeneity that characterizes these networks. Future studies should target specific brain regions or networks to deepen our understanding of their mechanisms and contributions to SCI‐related changes.

## 5 CONCLUSION

In summary, the results of our study demonstrate that patients with CSCI exhibit reduced efficiency in SC networks, while patients with ICSCI display enhanced local connectivity in FC networks, along with decoupling between SC and FC networks. Furthermore, changes in these network metrics are closely related to the sensory and motor functions of CSCI and ICSCI. Interestingly, differences in specific network metrics between the CSCI and ICSCI groups may act as objective markers for distinguishing between the two groups, aiding in the development of targeted rehabilitation and treatment strategies.

## AUTHOR CONTRIBUTIONS

All authors contributed to the study's conception and design. Material preparation, data collection, and analysis were performed by Beining Yang and Haotian Xin. The first draft of the manuscript was written by Beining Yang and revised by Nan Chen. All authors read and approved the submitted version.

## CONFLICT OF INTEREST STATEMENT

The authors have no conflict of interest to disclose.

## Data Availability

Research data are not shared.
